# Hip arthrogram parameters predict radiographic outcomes of patients with developmental dysplasia of the hip treated by closed reduction

**DOI:** 10.3389/fped.2023.1292928

**Published:** 2024-01-12

**Authors:** YunFei Tan, Wei Zhao, MinRong Wei, Yi He, HuaJun Deng, DaiWei Su, WuHui Zhu, YuQian Wu, Hao Shen, YiQiang Li

**Affiliations:** ^1^ Department of Pediatric Orthopaedics, Liuzhou Hospital of Guangzhou Women and Children's Medical Center, Liuzhou, China; ^2^ Department of Pediatric Orthopaedics, Guangzhou Women and Children’s Medical Center, Guangzhou Medical University, Guangzhou, Guangdong, China

**Keywords:** developmental dysplasia of the hip, closed reduction, hip arthrogram, residual acetabular dysplasia, avascular necrosis of the femoral head

## Abstract

**Objective:**

This study aimed to investigate the relationship between intraoperative hip arthrogram parameters and residual acetabular dysplasia (RAD) and avascular necrosis of the femoral head (AVN) in children with developmental dysplasia of the hip (DDH) treated by closed reduction.

**Methods:**

We retrospectively analyzed the data of 102 patients (110 hips; mean age, 14.6 months ± 4.7 months) with DDH treated by closed reduction. A hip arthrogram was routinely performed during the operation. The femoral head coverage rate (FHC), medial pool distance of the hip (MPD), labral inversion, and reduction quality classification were evaluated under the hip arthrogram. The presence of RAD and AVN was assessed on radiographs at the last follow-up. The relationship between each arthrogram parameter and RAD as well as AVN was investigated using a *t*-test, chi-square test, and logistic regression.

**Results:**

The overall FHC and medial pool distance of the hip (MDP) averaged 42.2% ± 12% and 8.1% ± 11.7%, respectively. There were 80 hips (72.7%) with labral inversion and 30 hips (27.2%) without. The reduction quality was type A in 57 hips (51.8%), type B in 28 hips (25.4%), and type C in 25 hips (22.7%). A total of 32 hips (29%) were in the RAD group, and 78 hips (71%) were in the recovered group according to whether pelvic osteotomy was performed or not and according to the last Severin grade. The FHC was significantly higher in the recovered group than that in the RAD group (*P* = 0.014). No significant difference was observed in sex, age at reduction, side, preoperative acetabular index, International Hip Dysplasia Institute classification, follow-up time, quality of reduction, MDP, and proportion of labral inversion between the recovered and RAD groups. Logistic regression analysis showed that only the FHC was a risk factor for RAD. The incidence of AVN above type II was 11.8% in this group of patients, and the incidence of AVN was significantly higher in patients with labral inversion (23.2%) than that in those without (7.5%; *P* = 0.041). Logistic regression analysis showed that labral inversion was a risk factor for AVN.

**Conclusion:**

The FHC measured under arthrogram can predict the occurrence of RAD after closed reduction of DDH, whereas MDP, reduction quality classification, and labral inversion are of little significance. Labral inversion is a risk factor for AVN.

## Introduction

Developmental dysplasia of the hip (DDH) is a common lower limb disorder in children, which includes complete hip dislocation, subluxation, and acetabular dysplasia (1). The incidence of DDH per 1,000 live births ranges from 0.06 in Africans in Africa to 76.1 in Native Americans (2) Generally, in most of the patients with DDH, only acetabular dysplasia or hip instability is present at birth, and hip dislocation gradually appears afterward if the diagnosis and treatment are delayed. The key to the treatment of DDH lies in early stable concentric reduction, promotion of acetabular and femoral head development, and avoidance of avascular necrosis of the femoral head (AVN) (3). At present, closed reduction and cast immobilization under general anesthesia is a common treatment for children over 6 months of age with DDH (4, 5). AVN and residual acetabular dysplasia (RAD) are the most comment complications in patients with DDH following treatment. The reported rates of AVN in patients with DDH undergoing closed reduction range from 0% to 67% (6–8).

RAD is another common complication in patients with DDH following closed reduction, and more than one-third of treated DDH patients have been reported with RAD (9, 10). It is well known that RAD may lead to abnormal gait, hip pain, and an increased rate of secondary osteoarthritis in adulthood (10, 11). Additionally, Ren et al. (12) reported that an abnormal relative position of the acetabulum–femoral head caused by hip dysplasia could accelerate the closure of the femoral head growth plate in immature female patients.

Hip arthrogram is a common method used to assist reduction and assess the quality of reduction when closed reduction is performed for DDH. In previous studies, some investigators used some intraoperative arthrogram indicators, such as medial pool distance of hip (MDP) (13–17), femoral head coverage rate (FHC) (18, 19), and labral inversion, to evaluate the quality of reduction and predict the outcome after closed reduction of DDH (19). However, the application of hip arthrogram in DDH is very controversial because of the different quality of arthrogram by different doctors and the differences in the judgment of arthrogram images. In this study, we retrospectively analyzed the clinical data of children with DDH treated with closed reduction in our institution to investigate the relationship between intraoperative arthrogram parameters and RAD and AVN after closed reduction in children with DDH.

## Materials and methods

The clinical data of children with DDH treated with closed reduction and cast immobilization were retrospectively analyzed from 2014 to 2020 in Guangzhou Women and Children's Medical Center and Liuzhou Hospital of Guangzhou Women and Children's Medical Center. The study was approved by the Ethics Committee of our hospital (NO: 2019-052).

Inclusion criteria were as follows: (1) diagnosed as DDH; (2) aged more than 6 months; and (3) treated with closed reduction and cast immobilization. Exclusion criteria were as follows: (1) failure of closed reduction or redislocation after successful reduction; (2) follow-up time less than 24 months, incomplete imaging data; and (3) combined with cerebral palsy, tethered cord, myelomeningocele, polyarticular contracture, and other neuromuscular diseases.

### Demographic indicators

A total of 102 patients (110 hips) met the inclusion criteria in this study, of whom 94 were females (92.1%) and 8 were males (7.8%) [60 left (58.8%), 34 right (33.3%), and 8 bilateral (7.8%)].

### Treatment and hip arthrogram

All children were treated with closed reduction and cast immobilization. Closed reduction was performed under general anesthesia with an intraoperative arthrogram at the time of reduction. During reduction, percutaneous transection of the adductor muscle was performed if the adductor muscle was tense and blocked the reduction. During radiography, the child was placed in a supine position with hip flexion and abduction and was punctured behind the adductor muscle into the hip capsule. Subsequently, 1.0–1.5 ml of contrast agent (iopromide) was injected to determine the contrast agent development and fully fill the joint capsule under fluoroscopy. At this point, the femoral head was reduced under traction, and the positional relationship between the femoral head and the acetabulum was reconfirmed by fluoroscopy. Cast immobilization of this position was maintained if the reduction was successful, and if it failed, open reduction was performed instead. The patients whose parameters of imaging of the arthrogram can not be measured were excluded from this study. The patients returned to the hospital 6 weeks after surgery to replace the cast, and the total time of cast immobilization was 3–4 months. The cast was then removed and switched to brace fixation for another 3 months.

### Preoperative imaging parameters

Before treatment, the degree of hip dislocation was classified according to the International Hip Dysplasia Institute (IHDI) criteria on AP pelvic radiographs (20). The appearance of the ossification nucleus of the femoral head was evaluated on AP pelvic radiographs. Acetabular index (AI) (21, 22) was also measured preoperatively on AP pelvic radiographs.

### Intraoperative arthrogram parameters

**Figure 1:**
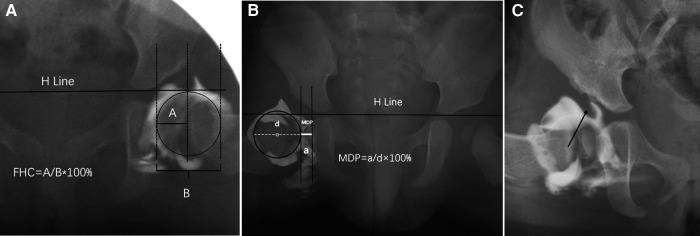
Measurement of femoral head coverage rate (FHC) (**A**) and MDP (**B**) on arthrogram. (**C**) indicates labral inversion.

On the arthrogram images, FHC and MDP were measured, and the presence or absence of labral inversion was also evaluated (Figure 1). The evaluation method was as follows: FHC is the percentage of the femoral head covered by the acetabulum on the radiograph of the frog position (19). When FHC was measured, two perpendicular lines to the H line were made on the medial and lateral sides of the femoral head, respectively, and a vertical line perpendicular to the H line was made through the outer edge of the acetabulum. The FHC is the ratio of the distance of the femoral head located on the medial side of the acetabulum to the diameter of the femoral head. MDP is the width of the medial wall of the acetabulum and the medial femoral head relative to the diameter of the femoral head under the frog position (15). Labral inversion refers to a significant filling defect at the upper outer edge of the acetabulum under the arthrogram and is shown using a contrast agent.

**Figure 2:**
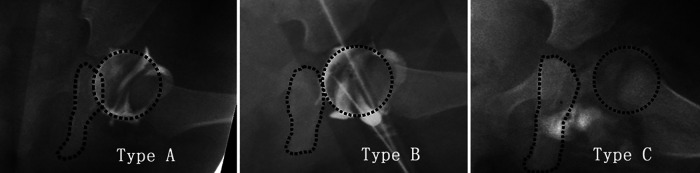
The classification of the quality of reduction. Type **A**: the medial aspect of the femoral head overlaps with the ischial ramus. Type **B**: the femoral head contacts the ischial ramus notch. Type **C**: the femoral head and ischial ramus notch separate.

The quality of reduction is divided into the following three types according to the relationship between the femoral head and the ischial ramus (19). Type A: the medial aspect of the femoral head overlaps with the ischial ramus. Type B: the femoral head contacts the ischial ramus notch. Type C: the femoral head and ischial ramus notch separate (19) (Figure 2).

### Postoperative follow-up parameters

AI central edge angle (CEA) and Reimer index (RI) (21, 22) were measured on AP pelvic radiographs at the last follow-up. All measurements involving x-rays were independently performed by two pediatric orthopedic surgeons, and their mean value was used for statistical analysis.

At the last follow-up, the treatment outcome was graded on AP pelvic radiographs according to Severin's method (23). Patients were divided into groups according to Severin's classification, namely, Severin grade I/II as the recovered group and Severin grade III/IV as the RAD group. Patients who underwent pelvic osteotomy correction for RAD were included in the RAD group regardless of the final outcome.

According to Kalamchi and MacEwen’s classification (24), AVN was assessed on an x-ray. Because type I AVN is temporary ischemic changes and can completely recover, we only counted types II, III, and IV when calculating AVN incidence (24). Two pediatric orthopedic doctors (SDW and TYF) judge AVN independently, respectively, and when the two judgments of AVN in a hip joint were inconsistent, three advanced pediatric orthopedic surgeons were required to make a discussion and decide the type.

### Statistical analysis

Statistical analysis was performed using SPSS 22.0 (SPSS, Chicago, IL, USA). Data included continuous numerical variables, frequencies, and percentages. The impact of arthrogram reduction quality, presence or absence of labral inversion, FHC, MDP on the outcome, and AVN was investigated using a *t*-test, chi-square test, and logistic regression analysis. Receiver operating characteristic (ROC) curve analysis was used to determine the cutoff values for positive factors to predict RAD. *P* < 0.05 was considered statistically significant.

## Results

A total of 102 cases (110 hips) were enrolled in this study, with a mean age at reduction of 14.6 months ± 4.7 months and a mean follow-up time of 58.5 months ± 24.8 months.

Before treatment, according to the IHDI classification, 15 hips (13.6%) were type II, 56 hips (50.9%) were type III, and 39 hips (35.5%) were type IV. Ossification nucleus of the femoral head was present in 84 hips (76.4%) and absent in 26 hips (23.6%) before reduction. The mean preoperative AI was 35.9° ± 4.5°.

Intraoperative arthrogram measured FHC and MDP, which averaged at 42.2% ± 12% and 8.1% ± 11.7%, respectively. There were 80 hips (72.7%) with labral inversion and 30 hips (27.2%) without. The reduction quality was type A in 57 hips (51.8%), type B in 28 hips (25.4%), and type C in 25 hips (22.7%).

AI, CEA, and RI were 20.7° ± 7.1°, 26° ± 10.6°, and 16.1% ± 12.4%, respectively, at the last follow-up. At the last follow-up, 88 hips (80%) were Severin grade I, 5 hips (4.5%) were in grade II, 16 hips (14.5%) were in grade III, and 1 hip (1%) was in grade IV. In total, 15 hips (13.6%) underwent pelvic osteotomy correction for RAD, 14 of which were Severin I and 1 Severin II at the last follow-up. Additionally. 32 hips (29%) were in the RAD group and 78 hips (71%) were in the recovered group according to whether pelvic osteotomy was performed or not and the last Severin grading.

**Figure 3:**
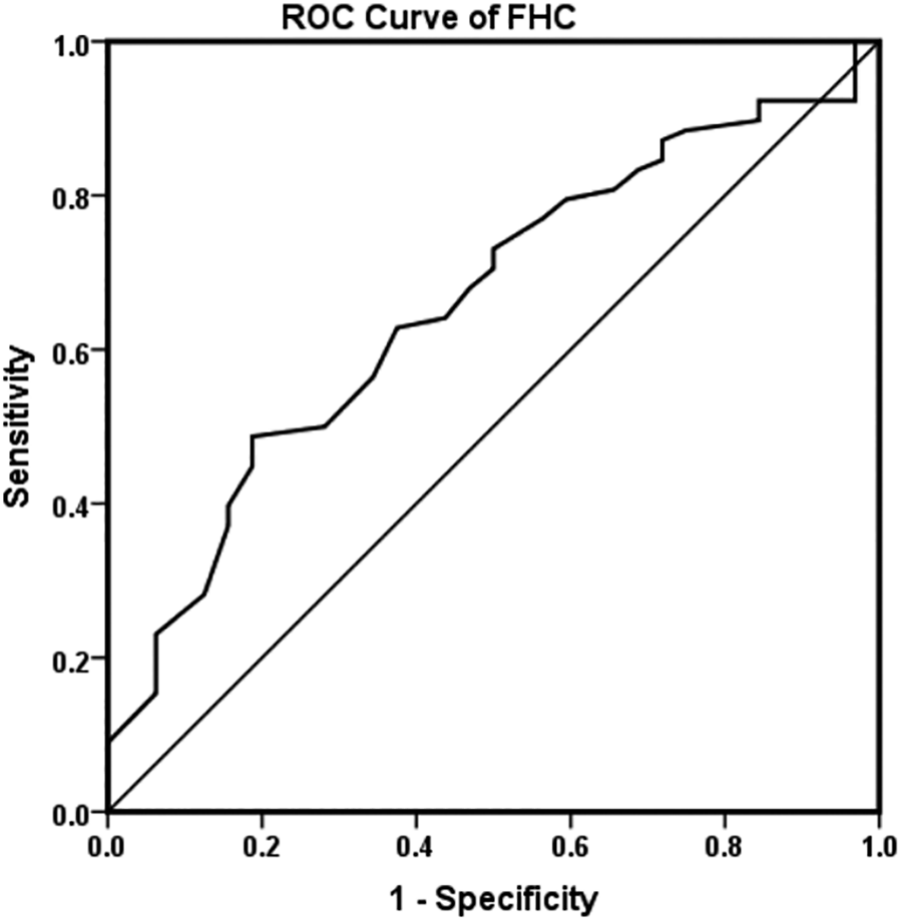
Receiver operating characteristic (ROC) curve for femoral head coverage rate (FHC).

FHC was significantly higher in the recovered group than in the RAD group (*P* = 0.014), and the incidence of RAD was significantly higher in patients without ossification nucleus of the femoral head before reduction (42.3%) than in patients with ossification nucleus of the femoral head (25%) (*P* = 0.047; Table 1). There was no significant difference in gender, age at reduction, side, preoperative AI, IHDI classification, follow-up time, quality of reduction, MDP, and proportion of labral inversion between the recovered and RAD groups (Table 1). Logistic regression analysis showed that only FHC was a risk factor for RAD (Table 2). ROC curve analysis indicated that 45.5% is the cutoff value of FHC to predict RAD (the area under the curve was 0.658; *P* = 0.009; Figure 3). In patients with a FHC <45.5%, 60.6% (40/66) of them had RAD, while in patients with a FHC >45.5%, 86.4% (38/44) of them recovered (*χ*^2 ^= 8.491, *P* = 0.004).

**Table 1:** Comparison of clinical data between the recovered group and the RAD group.

		Recovered group	RAD group	*χ*^2^/*t*/Z	*P*
Gender	Female	71 (70.3%)	30 (29.7%)	–	1.000^a^
	Male	7 (77.8%)	2 (22.2%)		
Age (months)		14.6 ± 4.8	15.2 ± 4.5	0.568	0.571
Side	Left	17 (28.3%)	43 (71.7%)	0.352	0.081
	Right	8 (23.5%)	26 (76.5%)		
	Bilateral	7 (43.8%)	9 (56.3%)		
Preoperative AI (**°**)		35.4 ± 4.2	37.1 ± 5.2	1.795	0.075
IHDI	II	4 (26.6%)	11 (73.4%)	1.194	0.232^b^
	III	17 (30.4%)	39 (69.6%)		
	IV	13 (33.3%)	26 (66.7%)		
Ossified nucleus of the femoral head	No	15 (57.7%)	11 (42.3%)	0.090	0.047
	Yes	63 (75.0%)	21 (25.0%)		
Follow-up time (months)		56.6 ± 23.3	65.2 ± 28.2	1.633	0.105
Reduction quality	Type A	36 (63.2%)	21 (36.8%)	1.597	0.111^b^
	Type B	23 (82.1%)	5 (17.9%)		
	Type C	19 (76.0%)	6 (24.0%)		
FHC (%)		44.0 ± 12.14	37.8 ± 10.7	2.502	0.014
MDP (%)		9.3 ± 12.6	5.4 ± 8.9	1.559	0.122
Labral inversion	No	9 (30.0%)	21 (70.0%)	1.0	0.183
	Yes	23 (28.7%)	57 (71.3%)		

^a^ Fisher's exact test.

^b^ Mann–Whitney *U* test.

AI, acetabular index; IHDI, International Hip Dysplasia Institute; FHC, femoral head coverage rate; MDP, medial pool distance of hip; RAD, residual acetabular dysplasia.

**Table 2:** Risk factors for RAD.

	Regression coefficient	Standard error	Wald value	*P*	RR	95% confidence interval for RR
FHC	−0.045	0.019	5.684	0.017	0.956	0.921, 0.992
Ossified nucleus of the femoral head	−0.807	0.482	2.799	0.094	0.446	0.174, 1.148

RR, relative risk; FHC, femoral head coverage rate; RAD, residual acetabular dysplasia.

In this study, 7 hips had type I AVN, 10 hips had type II AVN, and 3 hips had type III AVN. The overall incidence of AVN above type II was 11.8%. Patients with labral inversion had a significantly higher incidence of AVN (23.2%) than those without labral inversion (7.5%; *P* = 0.041; Table 3). No significant difference was observed in sex, age, side, IHDI classification, FHC, MDP, preoperative AI, and quality of reduction between patients with AVN and those without AVN (*P* > 0.05; Table 3). Logistic regression analysis showed that labral inversion was a risk factor for AVN (Table 4).

**Table 3:** Comparison of clinical data in patients with or without AVN.

		With AVN	No AVN	χ^2^/*t*/Z	*P*
		13 (11.8%)	97 (88.2%)		
Gender	Female	12 (11.9%)	89 (88.1%)	–	1.00^a^
	Male	1 (11.1%)	8 (88.9%)	–	1.00^a^
Age (months)		12.71 ± 5.30	5.23 ± 4.46	1.702	0.092
FHC (%)		45.0 ± 13.54	37.8 ± 10.7	0.407	0.685
MDP (%)		8.40 ± 14.71	8.0 ± 11.08	0.610	0.543
Preoperative AI (°)		34.66 ± 5.76	37.1 ± 5.2	0.430	0.675
Follow-up time (months)		55.69 ± 28.62	59.92 ± 24.29	1.807	0.497
Reduction quality	Type A	8 (14%)	49 (86%)	0.802	0.423^b^
	Type B	3 (10.7%)	49 (86%)		
	Type C	2 (8%)	23 (92%)		
Labral inversion	No	6 (7.5%)	74 (92.5%)	5.248	0.041
	Yes	7 (23.3%)	23 (76.7%)		
IHDI	II	3 (20%)	12 (80%)	0.741	0.459^b^
	III	6 (10.7%)	50 (89.3%)		
	IV	4 (10.3%)	35 (89.7%)		
Ossified nucleus of the femoral head	No	3 (11.5%)	23 (88.5%)	0.003	1.00^a^
	Yes	10 (11.9%)	74 (88.1%)		
Side	Left	9 (15%)	51 (85%)	–	0.65^a^
	Right	3 (8.8%)	31 (91.2%)		
	Bilateral	1 (6.3%)	15 (93.8%)		

^a^ Fisher's exact test.

^b^ Mann–Whitney *U* test.

AVN, avascular necrosis of the femoral head; FHC, femoral head coverage rate; MDP, medial pool distance of hip; AI, acetabular index; IHDI, International Hip Dysplasia Institute.

**Table 4:** Risk factors for AVN.

	Regression coefficient	Standard error	Wald value	*P*	RR	95% confidence interval for RR
Limbus inversion	−1.323	0.605	4.774	0.029	0.266	0.081, 0.873

RR, relative risk. AVN, avascular necrosis of the femoral head.

## Discussion

Hip arthrogram has important guiding significance for evaluating the reduction of the femoral head in patients with DDH treated by closed reduction. During hip arthrogram, a contrast agent was injected into the hip joint cavity to directly or indirectly observe the hip joint and its surrounding structures, including the acetabulum, acetabular labrum, femoral head, and round ligament (15). At present, different parameters are used under hip arthrogram to evaluate the quality of reduction and predict RAD (19); however, there are still controversies on the use of these parameters for the prediction of RAD.

**Figure 4:**
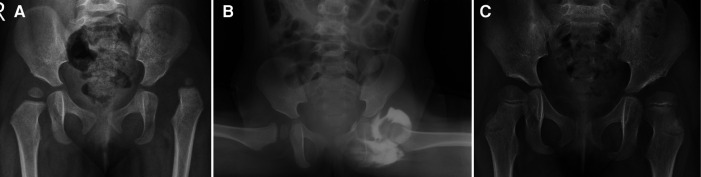
A 1-year-old female patient with left dysplasia of the hip (DDH) (**A**) treated by closed reduction. (**B**) The femoral head coverage rate (FHC) was 24%, there was a labral inversion, and the type of reduction quality was **B**. Three years after closed reduction, there was significant residual acetabular dysplasia (RAD) on the left side (**C**).

**Figure 5:**
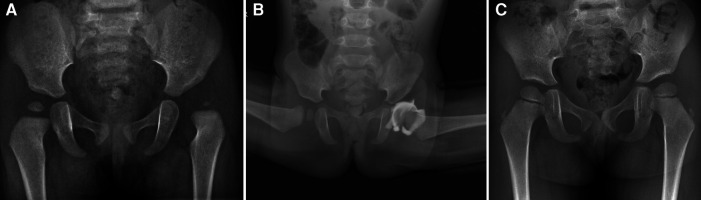
A 1-year-old female patient with left dysplasia of the hip (DDH) (**A**) treated by closed reduction. (**B**) The femoral head coverage rate (FHC) was 59%, there was no labral inversion, and the type of reduction quality was A. Three years after closed reduction, the left hip achieved a satisfactory outcome (**C**).

This study showed that FHC could predict the incidence of RAD after closed reduction of DDH. FHC refers to the proportion of the femoral head covered by the acetabulum and reflects the depth of the femoral head into the acetabulum (18). RAD is a common deformity following closed reduction of DDH. In this study, FHC in the recovered group (44.0% ± 12.14%) was significantly greater than that in the RAD group (37.8% ± 10.7%), and logistic regression analysis also confirmed that FHC was a risk factor for RAD. Some scholars have previously investigated the relationship between FHC and RAD and came to similar results as the present study. Zhang et al. (19) retrospectively analyzed the clinical data of 126 children (139 hips), with DDH treated by closed reduction and who underwent hip arthrogram under general anesthesia, and measured FHC. The results showed that FHC on the primary hip arthrogram was significantly higher in the non-RAD group (51.2% ± 15.3%) than that in the RAD group (28.5% ± 15.9%). Terjesen and Horn (25) retrospectively analyzed the clinical data of 49 patients (52 hips) with DDH and found that RAD significantly increased in patients with low FHC in the first year after reduction. Although FHC has an important value for predicting RAD in patients with DDH following closed reduction, the application of this parameter should be cautious. In the present study, the incidence of RAD was 60.6% in patients with FHC <45.5% (Figure 4), while it was 13.6% in patients with FHC >45.5%. It seems that it is more accurate when we use FHC > 45.5% to predict non-RAD (Figure 5).

This study did not find a significant correlation between MDP and RAD after closed reduction of DDH. There was also no significant difference in MDP between the recovered and RAD groups. Many researchers have previously investigated the use of MDP in hip arthrogram. Yuan et al. (26) investigated the clinical data of 173 patients (187 hips) with DDH treated with closed reduction and measured MDP to predict reduction failure. They showed that the risk of reduction failure was significantly increased when MDP was >6 mm. Race and Herring (13) retrospectively investigated data of 48 DDH patients (59 hips) who underwent closed reduction, in whom MDP values could be measured in 52 hips, and their results showed that MDP > 7 mm significantly increased the occurrence of RAD. The result of our study was different from those of Race and Herring (13); however, the sample size of this study is twice that of Race and Herring (102 patients with 110 hips), and our conclusions may be more reliable. We believe that there are several reasons why MDP has little reliability in predicting RAD. First, there are differences in the quality of arthrogram and the dosage of contrast agent among different surgeons, which directly affect the measurement of MDP. The second is that the MDP measures the width of a potential cavity on the medial side of the hip joint, and how much contrast agent it accommodates directly reflects the magnitude of the MDP value. However, when the hip joint is abducted at different angles, the medial space changes, resulting in the change of contrast agent filling, thus affecting the measurement of MDP. Third, MDP measurements require identification of the medial wall of the acetabulum and the medial aspect of the femoral head; however, there are differences in understanding of the medial wall of the acetabulum under contrast between different measurers. It can be seen that the measurement of MDP is affected by many factors, which makes the accuracy and repeatability of its measurement low.

This study did not find a significant correlation between labral inversion and RAD after closed reduction of DDH. Eighty hips (72.7%) had labral inversion in this study; however, the incidence of RAD in patients with labral inversion was not significantly different from that in patients without labral inversion. Previous studies have also shown that most DDH patients with closed reduction have (19, 27) labral inversion, although residual labral inversion increases the risk of RAD. Kaneko et al. (27) treated 40 patients (42 hips) with DDH with closed reduction and confirmed the presence or absence of residual labral inversion by MRI immediately after surgery and at 5 weeks after surgery, and the results showed that labral inversion was present in 40 hips (95.2%), but only 19 hips still had residual labral inversion at 5 weeks after surgery. The incidence of RAD was significantly higher in patients with residual labral inversion (84.2%) than in those without residual labral inversion (34.8%) at 5 weeks after surgery. Zhou et al. (28) also showed that labral inversion affects acetabular development. This is somewhat different from our findings, which may be related to the different timing of assessing labral inversion in each study. This study evaluated labral inversion only intraoperatively, whereas the study by Kaneko et al. (27) was performed immediately postoperatively and at 5 weeks postoperatively. Previous studies have shown that the inverted labrum could be remodeled after reduction. Fu et al. (29) investigated the morphology of the labral acetabular cartilage complex (LACC) on MRI images in 103 DDH patients treated with closed reduction, and they found that the morphology of the LACC would be shaped after closed reduction and some patients with labral inversion could become labral eversion. However, in patients with labral inversion initially, 58.8% of patients eventually had residual labral inversion (29). In addition, Liu et al. (30) also showed labral inversion in some patients gradually shaped to labral eversion after closed reduction, whereas labral inversion persisted in other patients with, but became a thin layer of fibrous tissue. It can be seen that labral inversion on intraoperative arthrogram does not accurately predict RAD and only labral inversion that persists postoperatively may be a risk factor for RAD.

This study did not find a significant correlation between reduction quality classification and RAD after closed reduction of DDH. The incidence of RAD with reduction quality A, B, and C was 36.8%, 17.9%, and 24.0%, respectively, and there was no statistically significant difference among the three groups. Zhang et al. (19) were the first to propose this classification of reduction quality in patients with DDH treated by closed reduction. Their findings also showed that the reduction quality was not a risk factor for RAD. The reason for this may be related to the defects in this classification. In this classification, type B is contact between the femoral head and the notch of the ischial ramus. However, it is difficult to clearly define how the femoral head contacts the ischial ramus under arthrogram, resulting in difficulty in classification.

This study indicated a significant increase in the incidence of AVN if the intraoperative arthrogram suggested labral inversion. The incidence of AVN was 11.8% in this group and 23.3% in patients with labral inversion, which was significantly higher than that in patients without labral inversion (7.5%). There was no significant difference in other arthrogram parameters (FHC, MDP, reduction quality) between patients with AVN and those without AVN. Currently, there are few studies on arthrogram and AVN incidence. Only Khoshhal et al. (31) randomly divided 85 patients (124 hips) with DDH into 2 groups, wherein 48 patients (79 hips) underwent hip arthrogram during closed reduction and 37 patients (45 hips) did not undergo arthrogram. The results showed that the incidence of AVN in the arthrogram group was significantly lower than that in the non-arthrogram group. In addition, labral inversion and distance from the ilium to the cartilage surface of the femoral head (greater than 4 mm) were risk factors for AVN in the arthrogram group. Khoshhal et al.'s findings were partially similar to our findings, both suggesting that labral inversion is a risk factor for AVN, but our study suggests that MDP is not a risk factor for AVN. Therefore, the relationship between intraoperative arthrogram parameters and the occurrence of AVN needs further study.

It should be noted that there are some limitations in this study. First, this is a retrospective study. Second, although the mean follow-up time was 59.2 months, it ranged from 24 to 123 months. As we know, the hip develops with age, as the rate of RAD may decrease in patients with a longer follow-up time, which could produce some bias to the outcome of the study. Additionally, long-term complications associated with hip dysplasia, such as femoral head necrosis, can manifest several years after the reduction procedure. Due to the restricted duration of the study's follow-up, it may be challenging to detect all instances of femoral head necrosis, which may influence the results of the study.

## Conclusions

A hip arthrogram has important guiding significance for evaluating the reduction of the femoral head in closed reduction of DDH. Arthrogram FHC can predict the occurrence of RAD after closed reduction of DDH, while MDP, reduction quality classification, and labral inversion are of little significance for predicting RAD after closed reduction of DDH. Labral inversion is a risk factor for AVN.

## Data Availability

The datasets presented in this study are available from the corresponding author.
